# Time-Resolved Cell Culture Assay Analyser (TReCCA Analyser) for the Analysis of On-Line Data: Data Integration—Sensor Correction—Time-Resolved IC_50_ Determination

**DOI:** 10.1371/journal.pone.0131233

**Published:** 2015-06-25

**Authors:** Julia Lochead, Julia Schessner, Tobias Werner, Stefan Wölfl

**Affiliations:** 1 Institute for Pharmacy and Molecular Biotechnology, Heidelberg University, Heidelberg, Germany; 2 Institute for Analytical Chemistry, Mannheim University of Applied Sciences, Mannheim, Germany; Massachusetts Institute of Technology, UNITED STATES

## Abstract

Time-resolved cell culture assays circumvent the need to set arbitrary end-points and reveal the dynamics of quality controlled experiments. However, they lead to the generation of large data sets, which can represent a complexity barrier to their use. We therefore developed the Time-Resolved Cell Culture Assay (TReCCA) Analyser program to perform standard cell assay analyses efficiently and make sophisticated in-depth analyses easily available. The functions of the program include data normalising and averaging, as well as smoothing and slope calculation, pin-pointing exact change time points. A time-resolved IC_50_/EC_50_ calculation provides a better understanding of drug toxicity over time and a more accurate drug to drug comparison. Finally the logarithmic sensor recalibration function, for sensors with an exponential calibration curve, homogenises the sensor output and enables the detection of low-scale changes. To illustrate the capabilities of the TReCCA Analyser, we performed on-line monitoring of dissolved oxygen in the culture media of the breast cancer cell line MCF-7 treated with different concentrations of the anti-cancer drug Cisplatin. The TReCCA Analyser is freely available at www.uni-heidelberg.de/fakultaeten/biowissenschaften/ipmb/biologie/woelfl/Research.html. By introducing the program, we hope to encourage more systematic use of time-resolved assays and lead researchers to fully exploit their data.

## Introduction

Cell culture systems are widely used for substance screening in drug discovery, the production of biotechnology products (enzymes, antibodies), the research of cellular functions or molecular pathways, and tissue engineering. The cell culture experiments are mostly monitored or evaluated through end-point assays, which are performed at one time point and generally cause irreversible damage to the samples. Time-resolved sensor assays have become easier to perform, without incurring additional costs, and present many advantages over end-point assays. They provide information about the dynamics of the observed phenomenon and allow the detection of reversible fluctuations that could have been otherwise missed, even if performing end-point assays at several time points. They also can read-out the measurement parameter before the experiment has begun and log the experimental procedure over time, thus offering quality control of the initial state of the experiment and its correct course and reproducibility. Moreover, through on-line monitoring it is possible to detect the time point of critical events and use the information for accurate planning of future experiment [1].

However, a major challenge brought by time-resolved sensor measurements is the proper analysis of the generated data sets especially in the case of high-throughput assays: even if the on-line methods are available and applied, they are often still analysed as end-point assays. This is due partly to the large size of the data sets that lead to time-consuming analysis, and partly to the unaccustomed and complex analysis possibilities offered by the time dimension. One example of this is the calculation of the slope of time-resolved curves, which provides information about how fast a measured analyte’s concentration changes over time, rather than just its actual value, reflecting for example cellular consumption or production.

A solution to improve the quality of time-resolved data analysis is an easy-to-use program facilitating tedious and recurring analytical processes, which can even include solutions for more complicated tasks. We therefore developed the freely accessible TReCCA Analyser: Time-Resolved Cell Culture Assay Analyser, program with a user friendly interface for the analysis of on-line data using the R programming language. The TReCCA Analyser was designed to perform all the basic cell culture analysis steps, such as normalisation to a control condition, averaging and standard deviation calculation of technical replicates. It is implemented with a smoothing function that enables a slope calculation function. After the analysis is performed, the program automatically plots the results according to user-defined parameters in order to also take advantage of the convenience and speed of data representation using computer programming.

The time-resolved sensor data analysis challenges also prevail in the particular context of viability assays, where up to now single time points are used for IC_50_/EC_50_ determination [2]. While a dynamic IC_50_, covering the whole time course of the experiment, has been defined before [3, 4], only a few selected time points of the time-resolved experiment were taken and then analysed by fitting each curve individually. An easy-to-evaluate time-resolved IC_50_ generated through the fitting of every single time point of the viability assay would provide precise information about the change in IC_50_ over time and lead to a better understanding of toxicity mechanisms as well as a more accurate comparison of similar drugs. Therefore such a function was also implemented in the TReCCA Analyser.

The commercially available OxoDish (PreSens Precision Sensing GmbH, Germany) was used as on-line viability assay to illustrate the capabilities of the TReCCA Analyser. It is a classical cell culture 24-well plate in which an oxygen sensor has been embedded in the bottom middle of each well. The sensors are constituted of a polymer that prevents cell attachment, the dissolved oxygen measured in the medium therefore reflects the average consumption of the surrounding cells. These sensors are read out non-invasively through a SensorDish Reader (SDR) directly in the incubator. Each sensor contains a luminophore which is quenched in the presence of oxygen, leading to different intensities and life times of luminescence. The intensity of the light exciting each sensor is sinusoidally modulated so that the delay between the excitation and emission light, caused by the luminophore decay time or phase angle, can be measured and analysed to determine the oxygen content of the sensor. This system does not rely on the unstable intensity of the signal emitted by the sensor and thus can be used for a long time period without any effect of the quenching of the luminophore [5].

When measuring the read-out of each sensor of OxoDish without any content, the output value varies slightly from sensor to sensor in one plate (due to slight variability in the excitation signal and sensor geometry in each well) and also from plate to plate (due to exact calibration of each SDR and its position in the incubator). These technical complications often occur in high-throughput experimental set-ups, where it is more difficult to unify the output of many independent measurements. A common approach to correct sensors is to divide all the data by a control condition and multiply it by the target value. This would be the way of proceeding when a linear calibration relationship between the measured entity and output applies, but is inaccurate for all other relationships. However, as the correction that is actually required would be too complicated and time-consuming using traditional spread sheet applications, the error arising by performing a linear correction is usually accepted. For the OxoDish, the calibration relationship between the phase angle and the dissolved oxygen is complex [3], but we determined that it can conveniently be modelled by an exponential equation. A time-resolved logarithmic correction was therefore also implemented in the TReCCA Analyser for all sensors with an exponential calibration curve.

In this publication, we present the possibilities offered by the TReCCA Analyser to handle time-resolved data by evaluating the IC_50_ of a classical anti-cancer drug Cisplatin, *cis*-[PtCl_2_(NH_3_)_2_] [6] on MCF-7 cells, a human breast carcinoma cell line [7], measuring dissolved oxygen with an OxoDish. The TReCCA Analyser facilitated the in-depth evaluation of the rate of dissolved oxygen change after Cisplatin exposure and its time-resolved toxicity.

## Materials and Methods

### Experimental design for the time-resolved IC_50_ of MCF-7 cells exposed to Cisplatin

#### Cell culture

The adherent MCF-7 cells (2012, CLS cell lines services GmbH, Germany, catalogue number 300273) were cultivated in Dulbecco’s modified eagle medium (DMEM) with 10% foetal calf serum (FCS) and without antibiotics, in a humidity controlled incubator with 5% CO_2_ and at 37°C. They were detached before reaching confluence using short washes with Dulbecco’s phosphate buffered saline (DPBS) and a trypsin substitute TrypLE Express (all solutions from Thermo Fischer Scientific Inc, United States), which was competitively blocked by full medium addition and removed through a centrifugation step (3 min, 1000 rpm).

#### Measurement settings

The oxygen was measured in percentage of air saturation, where 100% a.s. (air saturation) corresponds to the oxygen level in the air. The measuring interval was set to 1 min for sensor calibration and to 10 min for the rest of the measurement. The lot number of the OxoDish OD-1309-01 was entered into the SDR’s software (SDR_v38, PreSens Precision Sensing GmbH, Germany) allowing for the general calibration of the plates performed by the company for the whole lot.

#### Sensor correction

The read-out of all the 24 sensors of two empty OxoDish was measured in the incubator for at least 10 time points after the read-out was stable (adaptation to temperature, oxygen and humidity conditions in the incubator).

#### Seeding

The MCF-7 cells (passage 39) were seeded in a volume of 500 μL at a density of 150 000 cells/well in order to obtain a significant oxygen reduction. Three wells of each plate were filled with medium only in order to control the oxygen level in the incubator. The medium was changed gently 24 h after seeding.

#### Cisplatin treatment

Fresh stock solutions of 5 mM and 1 mM Cisplatin (Sigma-Aldrich LLC, United States) were prepared in a 0.9% NaCl solution (B. Braun Melsungen AG, Germany). 24 h after medium change, different negligible volumes (30 μL or less) of the Cisplatin stock solutions were added to each well in triplicates (final concentrations: 300, 200, 100, 90, 80, 70, 60, 50, 40, 30, 20, 10, 0 μM).

### Program description

#### Programming environment and packages

The statistical framework R was chosen as programming environment [8], complemented with R packages for the fitting of sigmoid curves: drc [9] and the generation and customisation of the graphical user interface (GUI): gWidgets [10], gWidgetsRGtk2 [11], RGtk2 [12] and cairoDevice [13], which are dependent on the GTK+ widget library (http://www.gtk.org/). These resources are all freely available and compatible with Windows and Mac computers.

#### User interface

**Fig 1:**
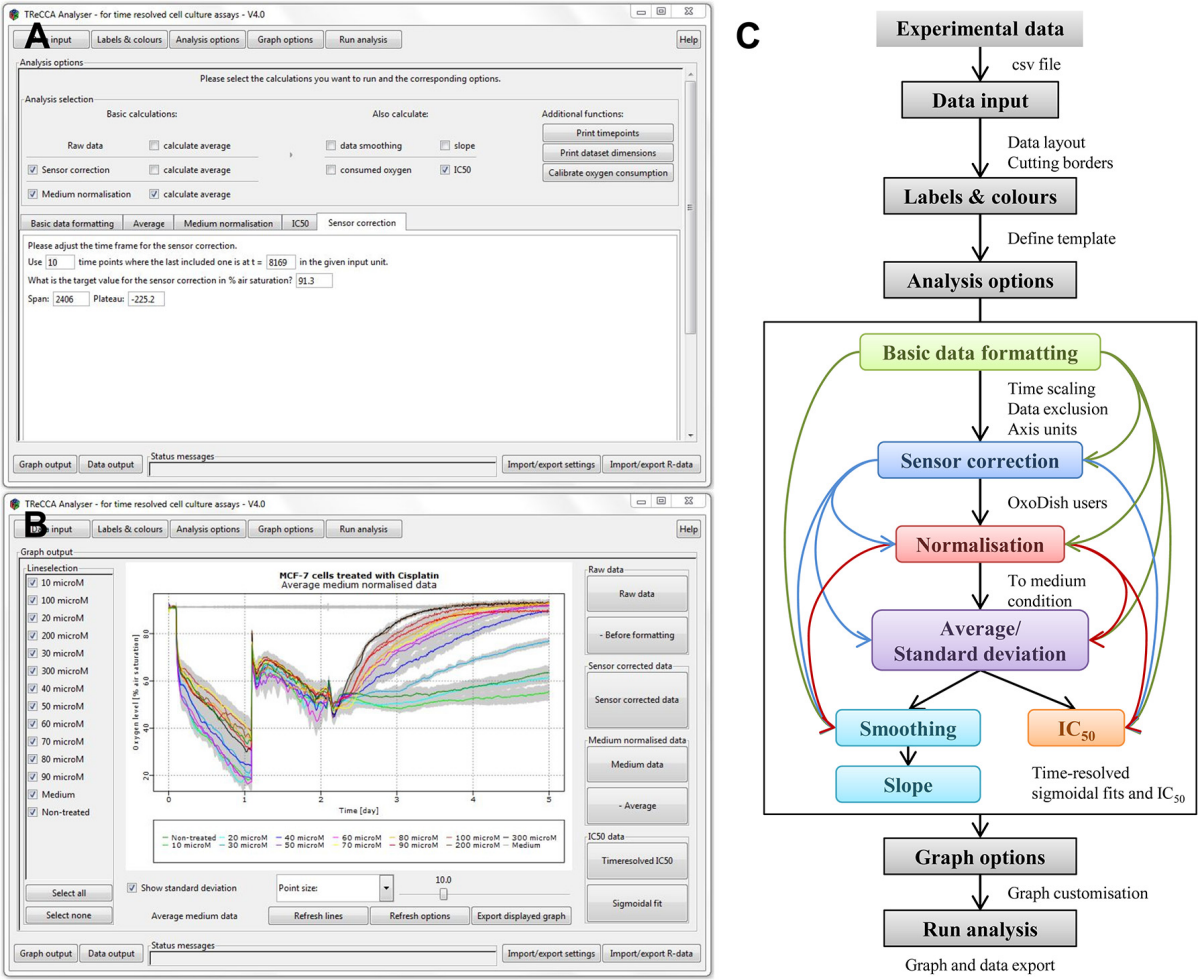
TReCCA Analyser user interface and analysis steps. Consecutive tabs are clicked through for data analysis and plotting. These include the “Analysis options” tab (A), where the analyses to be performed on the data are selected and their corresponding parameters entered, and the “Graph output” tab (B), where the graphs are visualised and can be further customised. These tabs can be seen at a higher resolution in S1 and S2 Figs. All the analysis steps are summarised in the program flow chart (C). The black rectangle encloses a more precise representation of the available analysis options. The black arrows depict the succession of steps that are performed in our example of the analysis of the effect of Cisplatin on MCF-7 cells, the coloured arrows depict alternative analysis flows.

Particular effort was put in designing a friendly user interface for the TReCCA Analyser. It was improved after being tested by 25 users for intuitiveness, clarity, and speed. Two screen shots of different tabs of the user interface, the “Analysis options” tab and the “Graph output” tab, are displayed in Fig 1A and 1B respectively.

#### Analysis flow

The flow chart in Fig 1C depicts the analysis flow of the TReCCA Analyser. The first step consists in importing the data into the program as a.csv file(s) in the “Data input” tab. The first column has to contain the time points of the experiment and the other columns the time-resolved data. The file import function can be customised to fit the experimental output files. In the “Labels and colours” tab, a template is filled with the experimental layout indicating which condition is to be found in each column of the data and how to plot it (colour, line type, legend). The template is a guide for the analysis, for example it determines which columns should be averaged as technical replicates. The analysis options and corresponding parameters, described more in detail in the next paragraphs, are selected from the “Analysis options” tab. Finally, the graph options are picked out from the “Graph options” and “Graph output” tab allowing the automatic customised creation of the graphs corresponding to each step of the analysis. These graphs, as well as.csv files containing all the analysis results, can then be exported by the user for further use through the “Graph output” and “Data output” tabs respectively. All of the settings can be saved for later reference and similar experiments. All the data can also be saved as an R-data file to allow a new customisation and exportation of the graphs without having to run the analysis again.

#### Analysis options

The analysis options available in the TReCCA Analyser are briefly presented in the following paragraph, more details are available in the S1 File (for the equations) and TReCCA Analyser user manual (www.uni-heidelberg.de/fakultaeten/biowissenschaften/ipmb/biologie/woelfl/Research.html).

Basic formatting: The time unit can automatically be converted by indicating the input unit and desired output unit, and time points at the beginning or the end of the measurement can be excluded. Moreover, the time frame of the data can be shifted (to set a different start experiment time) or the whole data can be scaled to another unit by multiplying it by a fixed factor.

Sensor correction: In the challenging case where the calibration relationship follows an exponential equation of the form *P* + *Se* ^*αy*^ (where *P* is the plateau, *S* the span and *α* an unknown factor), a sensor correction can be performed. This is the case with the OxoDish, although a Stern-Volmer relationship would be expected [14], as described in S3 and S4 Figs and S2 File. The data *x* is first converted to the correct logarithmic form *z* = ln((*x* − *P*)/*S*). Then the mean of each sensor *m*_*s*_ is calculated over the *n* time points (Eq A in S1 File), as well as the mean of the whole plate *m*_*p*_, which is the average of all the sensor means *m*_*s*_ for *s* number of sensors (Eq B in S1 File). The sensor corrected value *x*_*t*,*s*_ for each time point *t* and each sensor *s* is then corrected and converted back to logarithmic form (Eq 1), followed by the normalisation step of sensor correction (Eq C in S1 File).

$$x_{t,s}=P+Se^{\frac{m_{p}}{m_{s}}\;z_{t,s}}$$ (1)

Normalisation: Each data column can be normalised over time to a reference condition set at a target value, for example a non-treated condition being set to 100% of viability. First, the columns with the data of the normalisation condition are automatically detected through the template and their average at each time point *m*_*t*_ is calculated. Then, every single value is divided by its corresponding value *m*_*t*_ and multiplied by the target value *M* (Eq D in S1 File), thereby setting the normalisation conditions to the target value and shifting the other conditions.

Average and standard deviation: The average and the standard deviation of technical replicates are automatically determined for each time point, according to either the name, number or colour given to each column in the template (Eq E in S1 File).

Smoothing: The data smoothing is performed by replacing a measured data point *x*_*t*_ by the average of n neighbouring data points around that time point (Eq F in S1 File). This transformation leads to the loss of (*n* − 1)/2 data points at the beginning and at the end of the measurement and *n* necessarily has to be an uneven number.

Slope calculation: A linear fit function built in R is applied to each smoothed data point and *n* smoothed data points on either side (Eq G in S1 File) to determine the slope *m* and the corresponding residual (which is displayed as a grey shade behind the graphs).

Time-resolved IC_50_: The IC_50_/EC_50_ of a compound is determined based on any of the previous time-resolved data. First, the time frame that should be used for the IC_50_ calculation is set, as well as how regularly the fittings should be performed. The user has to assign concentrations to the labels of the template, and the different concentrations are then fitted automatically and over time. Four different log-logistic model functions, each varying by their number of parameters, are available to perform the sigmoid fit (Eqs H, I and J in S1 File), the four logistic being the most commonly applied one (Eq 2)

$$f\left(x,\;\left(b,c,d,e\right)\right)=\frac{d-c}{1+e^{\left(b\left(\log\left(x\right)-\log\left(e\right)\right)\right)}}+c$$ (2)

## Results

### Time-resolved IC_50_ of MCF-7 cells exposed to Cisplatin

To illustrate the capabilities of the TReCCA Analyser, we analysed the oxygen levels in the media of MCF-7 cells exposed to different Cisplatin concentrations following the steps depicted in the flow chart in Fig 1C and going through the main analysis functions of the program. The data presented here originates from one biological replicate; other independent replicates were however performed to confirm the results. The publication graphs can be reproduced by following the instructions of the TReCCA tutorials 1 and 2, which also provide the raw data (www.uni-heidelberg.de/fakultaeten/biowissenschaften/ipmb/biologie/woelfl/Research.html).

#### Basic formatting

**Fig 2:**
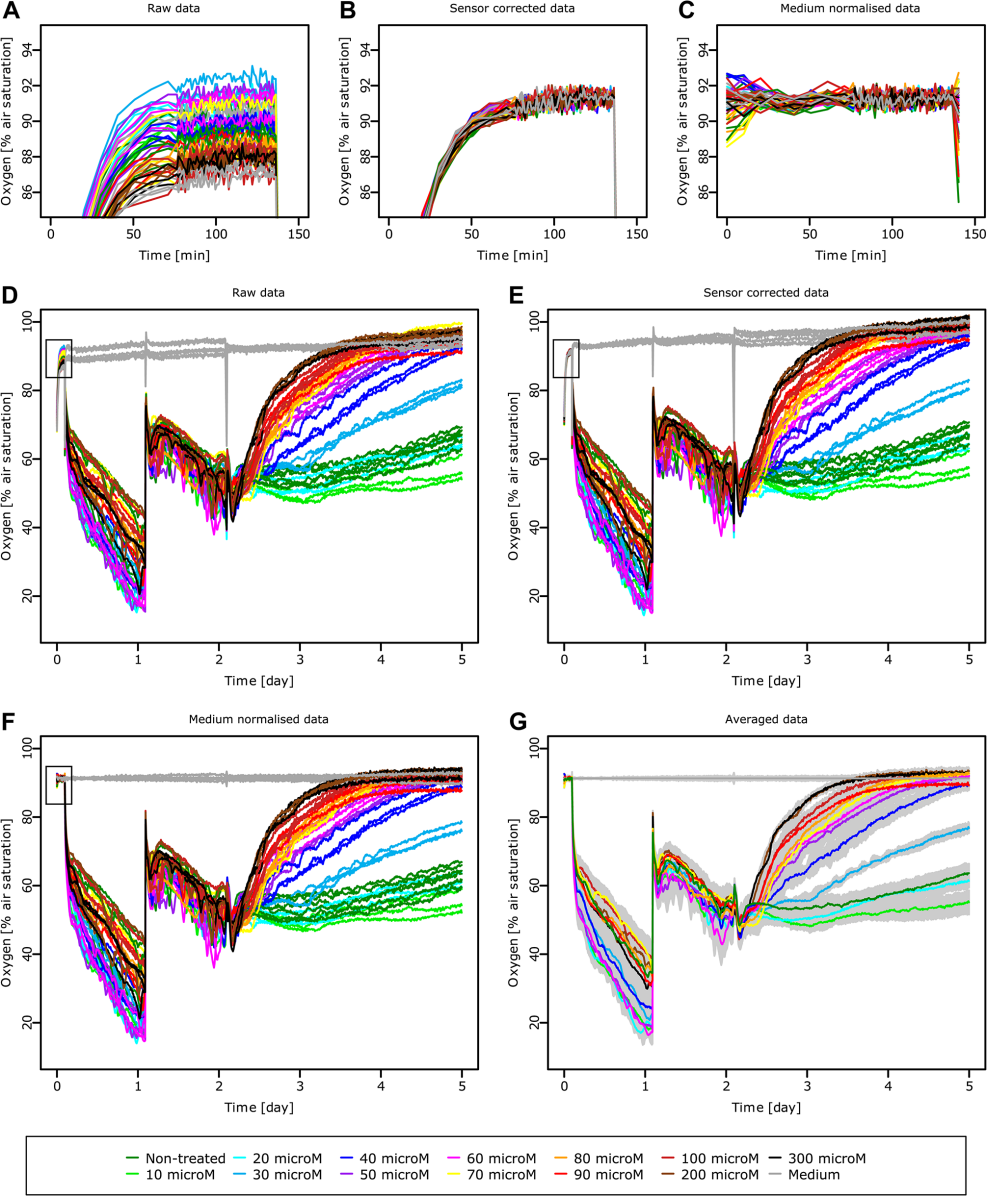
Medium oxygen level of MCF-7 cells when exposed to different concentrations of Cisplatin over time. All the sensors are measured empty for sensor calibration between minutes 0 and 136 (A), then a sensor calibration is performed (B), followed by normalisation to the wells containing only medium (C). The whole time frame of the experiment is visible in the raw data (D): MCF-7 cell seeding at 136 min, medium change at day 1.09 and Cisplatin addition at different concentrations at day 2.1. The sensor correction and the normalisation to the conditions containing only medium are applied to all the data (E, F respectively). The time points used for sensor correction/normalisation (A, B, C) are depicted as black rectangles (D, E, F respectively). The triplicates of the sensor corrected and normalised data are averaged (G) and their standard deviation is displayed as a grey shadow around each curve. The legend at the bottom of the figure is valid for all the subfigures, microM stands for micromolar. These graphs are displayed as they are produced by the TReCCA Analyser.

The MCF-7 and Cisplatin treatment data was extracted from the software provided with the SDR reader (SDR version v38), which is required for recording but does not enable adequate analysis of the data obtained. The TReCCA Analyser provides analysis options and plotting possibilities for the SDR data, but is also suitable for any other type of large time-resolved analytical measurements. The time scale was first converted from seconds to days. The last part of the graph is excluded, as after day 5 the viability of the controls decreases due to the lack of medium change. The raw data obtained after basic data formatting can be seen in Fig 2D. The cells seeded on the first day reduce the level of dissolved oxygen, which is replenished after medium change in the second day. The dissolved oxygen is then reduced again but this time in a more homogeneous way and to a lesser extent as the cell adhesion process does not interfere with the basal cell respiration levels. Starting on the third day, the dissolved oxygen level depends on the Cisplatin concentration: high Cisplatin concentrations lead to cell death and higher dissolved oxygen values. The cells in the non-treated condition reach confluence, the cell number and thereby the total oxygen consumption becomes constant as reflected by the stable dissolved oxygen values.

The data gathered on the first two days serves as quality control for the experiment: the basal cell respiration is as expected and reflects on the health of the cells; there are no outlier curves due to experimental errors or bacterial/yeast contaminations (that would have caused a sharp decrease in dissolved oxygen levels), the equipment is running correctly, the time points were respected exactly.

#### Standard data analysis

Sensor correction (recalibration and normalisation) was performed on the raw data as detailed in the S2 File, using 91.3% a.s. as the target value (Fig 2E), thereby homogenising the initial output as can be seen more precisely on a smaller oxygen scale (Fig 2A and 2B). For experiments only taking place on such a scale, the sensor correction can be critical for the distinction of different conditions (S5 Fig).

As the level of dissolved oxygen measured in wells containing only medium slightly increases over the course of the experiment, the sensor corrected data was normalised by setting the average of the control wells to 91.3% a.s. (Fig 2F). The media curves are consequently straight horizontal lines and the rest of the data is slightly shifted accordingly. On a smaller scale, the normalisation does not affect the stabilised calibration values (Fig 2C). The technical replicates of the normalised data are then averaged (Fig 2G). The corresponding standard deviations are displayed as grey shades, which is usually challenging for highly resolved data sets using common spread sheet applications.

#### Smoothing and slope calculation

**Fig 3:**
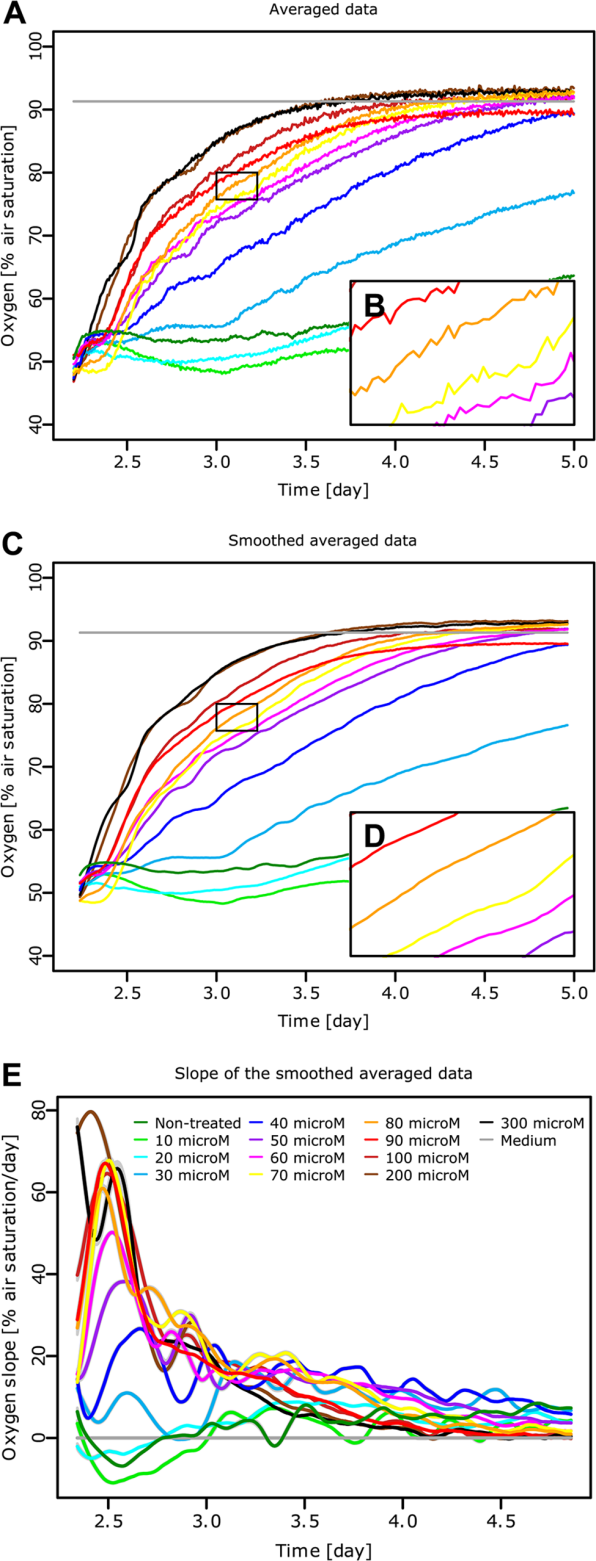
Smoothing and slope calculation. The oxygen levels in the media of MCF-7 cells exposed to different Cisplatin concentrations (as depicted in Fig 2G) between day 2.2 and 5 (A) are smoothed (C) by replacing each time point by the average of an 11 time point-neighbourhood. The smoothing can be seen more precisely on a bigger scale as it is the case for 75–80% a.s. and day 3–3.5 by comparing the raw data (B) to the smoothed data (D). The slope of each time point (E) is then obtained by performing a linear fit of each point and 15 points on either side of it. The residual error of the fit is displayed as a grey shadow around each curve (very small in this case). The legend in Fig 3E is valid for all the subfigures, microM stands for micromolar.

For the next analysis steps, we focus on the data after Cisplatin treatment between day 2.2 and 5 (Fig 3A). The time frame could have been shifted to start at the time of treatment but was left for clarity. In order to determine the slope of the curves over time, the averaged data is first smoothened for *n* = 11 (Fig 3C) as can be seen more precisely at a bigger scale (Fig 3B and 3D). The slope of the smoothed data is then determined for *n* = 15 (Fig 3E). It reveals that the amount of dissolved oxygen increases first and fastest for the highest Cisplatin concentrations, its rate of change reaches a maximum and then decreases slowly until stabilising. Lower Cisplatin concentrations follow the same reaction pattern, however the initial speed of change in respiratory behaviour is slower. The non-treated condition and the lowest Cisplatin concentrations have a constant oxygen consumption during the course of the experiment, as the slope is close to zero. The slope calculation allows to pin-point the exact time when the change of respiration is highest or constant; it also leads to a more accurate comparison of the different Cisplatin concentrations.

#### Time-resolved IC_50_ calculation

**Fig 4:**
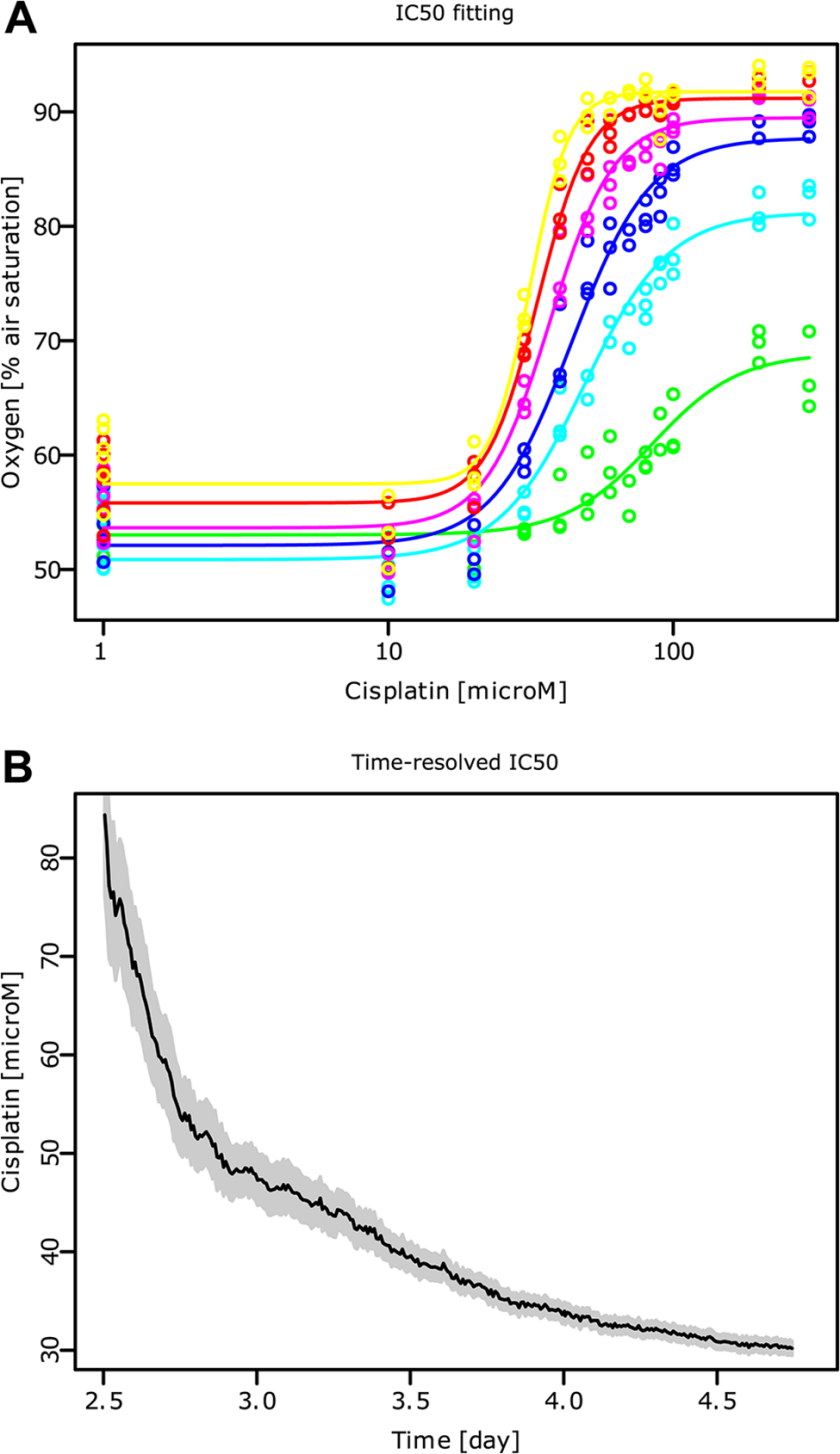
Time-resolved IC_50_ of MCF-7 cells exposed to Cisplatin. The oxygen level in the media of MCF-7 cells treated with different Cisplatin concentrations between days 2.2 and 5 (as depicted in Fig 3A) are fitted for each time point, as exemplified for day 2.50 (green), 2.89 (turquoise), 3.28 (blue), 3.66 (pink), 4.05 (red), 4.43 (yellow) (A). MicroM stands for micromolar. The IC_**50**_ over time (B) is then determined from the values of all the fits between days 2.5 and 4.7. The residual error of the fit is displayed as a grey shadow around each curve.

The four-parameter log-logistic model (Eq 2) is fitted to the averaged data for every time point between day 2.5 and 4.7 (Fig 4A). The parameter *e*, which is the IC_50_ value, is then plotted over time to display the time-resolved IC_50_ of Cisplatin (Fig 4B) which ranges from 80 μM at day 2.5, to 30μM at day 4.7. These values are in accordance with previously published data [15, 16] taking into account diverging protocol details (seeding density, Cisplatin solvant, viability test, time points). The longer the exposure to Cisplatin, the lower the concentration needed to reach 50% control viability. This decrease begins almost immediately, it is fast for short exposure times but levels out progressively, almost reaching a steady-state. Cisplatin has a rapid effect on cell respiration combined with a high cell permeability, its lack of stability over time could explain the levelling out.

## Discussion

The TReCCA Analyser was used to analyse the time-resolved toxicity of Cisplatin towards MCF-7 cells by measuring the level of dissolved oxygen in an OxoDish. It significantly reduced the effort and time needed for basic cell culture analysis steps and consequent graph plotting, as well as enabling a more thorough data analysis. The calculation of the slope of the data lead to the determination of the rates of dissolved oxygen change of the MCF-7 cells exposed to different Cisplatin concentrations and therefore to the observation of different reaction speeds to the drug and the detection of kinetic time points.

The sensor correction method improved the homogeneity of the OxoDish sensor read-outs, which could be crucial to detect different conditions in small scale experiments. The OxoDish has a vast field of applications where this sensor correction can also be applied, such as the monitoring of the oxygen consumption of bacterial cultures to study bioprocessing at a very small scale [17], the respiration of human spheroid cancer cells for tumour cell metabolism [18], the oxygen consumption of doliolids feeding at different food levels [19] or toxicity tests on zebrafish embryos [20]. Moreover, the logarithmic sensor recalibration can be applied to any other type of sensor where the relationship between the measured and output analyte is exponential.

The TReCCA Analyser also enabled the determination of the time-resolved IC_50_ of Cisplatin on MCF-7 cells, showing a fast onset of toxicity that begins to stabilise at the highest level 48 h after drug exposure. On top of providing information about how fast IC_50_ values are reached, the time-resolved IC_50_ could lead to a more accurate comparison of different drugs. It could be applied to a wide variety of time-resolved viability assays such as the non-toxic Alamar blue/resazurin assay, as performed in different cell lines and rat primary hepatocytes [21]; the measurement of intracellular adenosine 5′-triphosophate (ATP) concentrations using the firefly luciferase system, as applied to growing Escherichia coli [22]; the fluorescein diacetate assay (FDA), used to assess the cellular viability of plant cells [23]; and the MitoXpress assay, a phosphorescent oxygen-sensing probe, employed for on-line mammalian cell respiration monitoring [24].

The TReCCA Analyser can more generally be applied to any kind of time-resolved measurements, like on-line pH measurements [25], on-line glucose, ethanol and cell density measurements in the context of yeast fermentation [26], or enzyme activity kinetics [27] (Additional applications of the TReCCA Analyser are displayed in S6 Fig). On top of being convenient and fast (most analyses are performed in less than 15 minutes), the TReCCA Analyser leads to high accuracy, since the fixed formulas prevent human mistake and assure high reproducibility, as the exact settings of the analysis and plotting can be saved and reused or shared easily. The vast graph customisation possibilities ensure that the resulting graphs can be applied directly for presentation or publication purposes.

We hope that the TReCCA Analyser will help encourage scientists to work with big data sets without having to face the barrier of computer programming. Through the easy access to more complicated data analysis, more thorough data exploitation can be systematically performed. We are convinced that any continuous measurement that is translated to an electrical signal can benefit from this program. It is also completely open source allowing any scientist to modify it, adding functions that might be useful for further applications, not only in the field of cell culture assays but for any time-resolved sensor data analysis.

## Supporting Information

S1 Fig TReCCA Analyser “Analysis options” tab. Magnification of Fig 1A.
(TIF)

S2 Fig TReCCA Analyser “Graph output” tab. Magnification of Fig 1B.
(TIF)

S3 Fig Development of the OxoDish sensor correction method. The standard deviation of the read-out of the 24 sensors of one OxoDish is compared at different oxygen levels for the raw data, the data corrected by the classical multiplication/division correction method, and the logarithmic corrected method (Fig A). After the step 1 of sensor correction: recalibration, the differences between the incubator value and the average sensor-measured data is plotted for 3 independent OxoDish against the actual oxygen value (Fig B). % a.s. stands for percentage of air saturation and the error bars represent standard deviations.
(TIF)

S4 Fig OxoDish sensor correction testing. Read-out of 3 empty independent OxoDish, each containing 24 oxygen sensors, when placed in an oxygen-controlled incubator set consecutively at 91.3, 72.1, 48.1, 24.0 and 4.8% a.s. (dashed black horizontal lines). Using the TReCCA Analyser, the raw oxygen values given by all the sensors (Fig A) are made more homogeneous by step 1 of sensor correction: logarithmic recalibration (Fig B), and set to their target value by step 2 of sensor correction: linear normalisation (Fig C).
(TIF)

S5 Fig Application of the OxoDish sensor correction to cell seeding. Oxygen level in the media of the Neuroblastoma cell line IMR5/75 at different seeding densities before (Fig A) and after sensor correction (Fig B). The legend in the Fig B in S5 Fig is valid for both subfigures.
(TIF)

S6 Fig Additional applications of the TReCCA Analyser. The pH of the medium of HT29 cells (ATCC HTB-38) cells seeded at varying seeding densities on day 0 was measured continuously using the SDR and HydroDish system (PreSens Precision Sensing GmbH, Germany) (Fig A). On day 1, the medium was changed in each condition setting the pH back to its original level. The data was normalised by setting the medium condition (containing no cells) to pH 7.6 and the technical triplicates were averaged (Fig B). The levels of ROS (Reactive Oxygen Species) were continuously measured in IMR5/75 cells when exposed to tBHP (tert-Butyl hydroperoxide). The cells were seeded in a 96-well plate at 20 000 cells/well and after 24 h they were treated and stained with 5 μM of CellROX Deep Red Reagent (Thermo Fischer Scientific Inc, United States). The fluorescence was continuously measured according to the recommendations of the dye provider using the Safire plate reader (Tecan Trading AG, Switzerland). The raw data (Fig C) was averaged (Fig D) and smoothed (Fig E) using the TReCCA Analyser. The level of dissolved oxygen in the medium of HT29 three-dimensional structures was continuously measured using a 96-well plate containing oxygen sensors in each well (OxoPlate, PreSens Precision Screening GmbH, Germany) and the Infinite 200 Pro plate reader (Tecan Trading AG, Switzerland). On day 1, half of the medium was exchanged and different concentrations of Cisplatin (Sigma-Aldrich LLC, United States) were added to each well in sextuplates. The raw data (Fig F) was normalised to the conditions with no cells and averaged (Fig G). The time-resolved IC50 was determined (Fig I) by continuously fitting the data using the TReCCA Analyser, as exemplified for seven time points (Fig H).
(TIF)

S1 File TReCCA Analyser equations. The supplementary equations are grouped in this file.
(PDF)

S2 File Sensor correction for OxoDish. Details about the sensor correction method in the particular example of OxoDish use are presented in this file.
(PDF)
