# Supplementary figures and images for: Time-Resolved Cell Culture Assay Analyser (TReCCA Analyser) for the Analysis of On-Line Data: Data Integration—Sensor Correction—Time-Resolved IC50 Determination
Source: PLoS One. 2015 Jun 25;10(6):e0131233. doi: 10.1371/journal.pone.0131233 (PMC4482264; doi:10.1371/journal.pone.0131233)

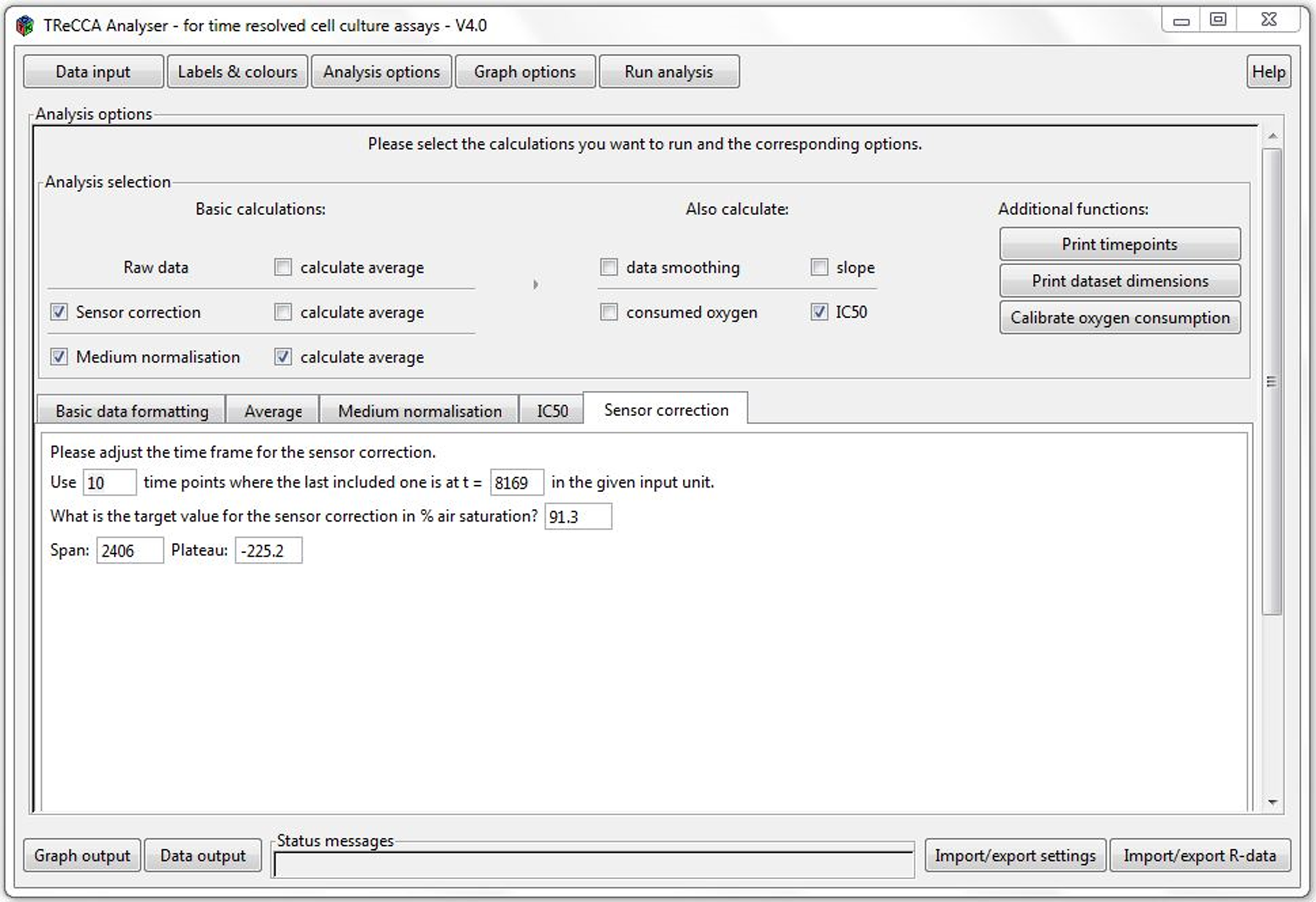

Supplement: S1 Fig — Magnification of Fig 1A. (TIF) [file pone.0131233.s001.tif]

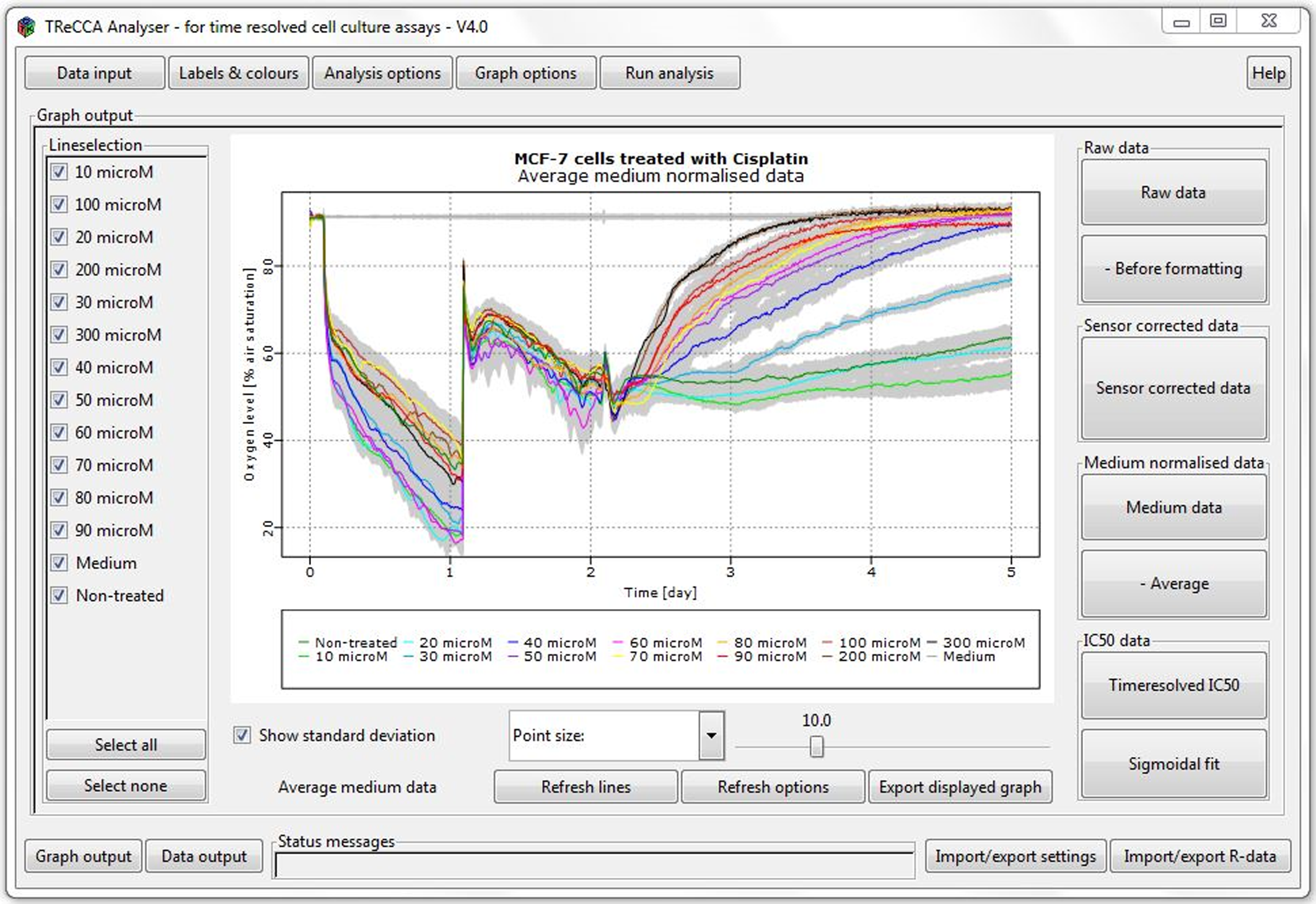

Supplement: S2 Fig — Magnification of Fig 1B. (TIF) [file pone.0131233.s002.tif]

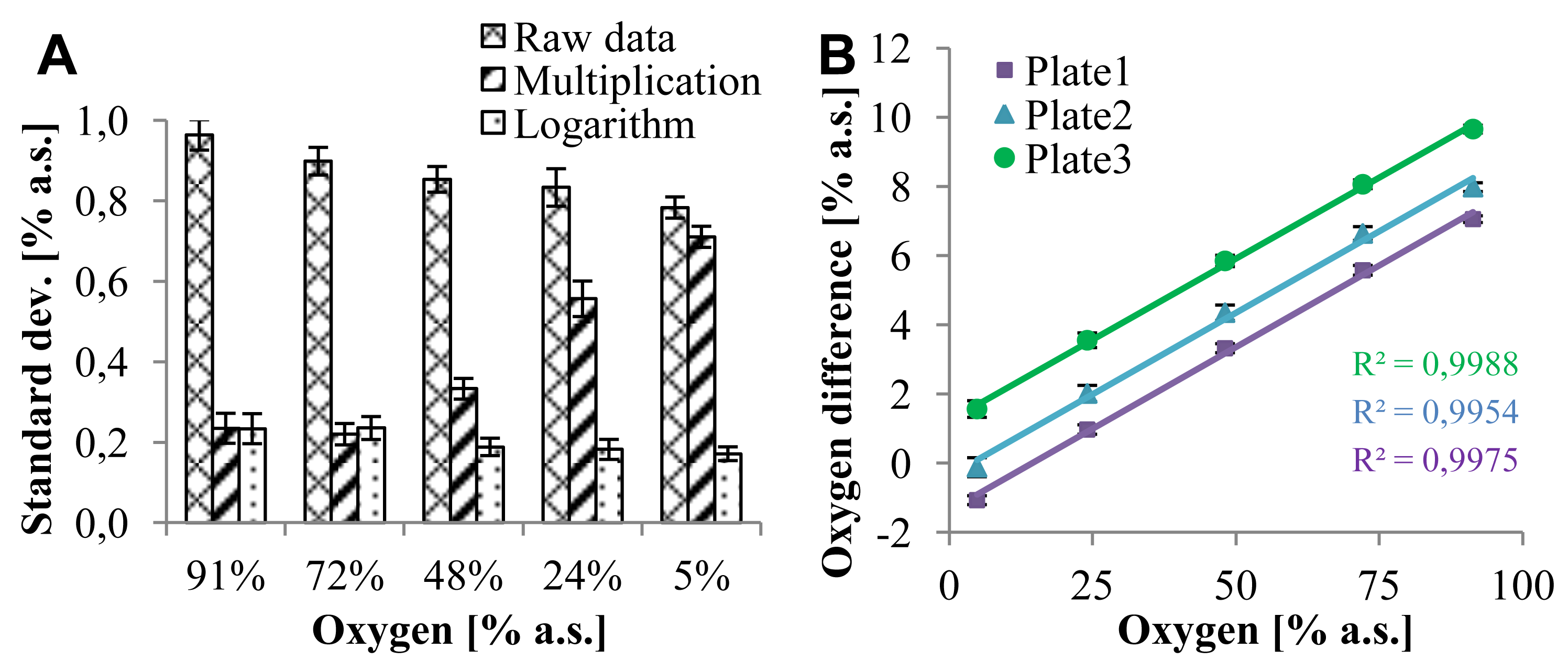

Supplement: S3 Fig — The standard deviation of the read-out of the 24 sensors of one OxoDish is compared at different oxygen levels for the raw data, the data corrected by the classical multiplication/division correction method, and the logarithmic corrected method (Fig A). After the step 1 of sensor correction: recalibration, the differences between the incubator value and the average sensor-measured data is plotted for 3 independent OxoDish against the actual oxygen value (Fig B). % a.s. stands for percentage of air saturation and the error bars represent standard deviations. (TIF) [file pone.0131233.s003.tif]

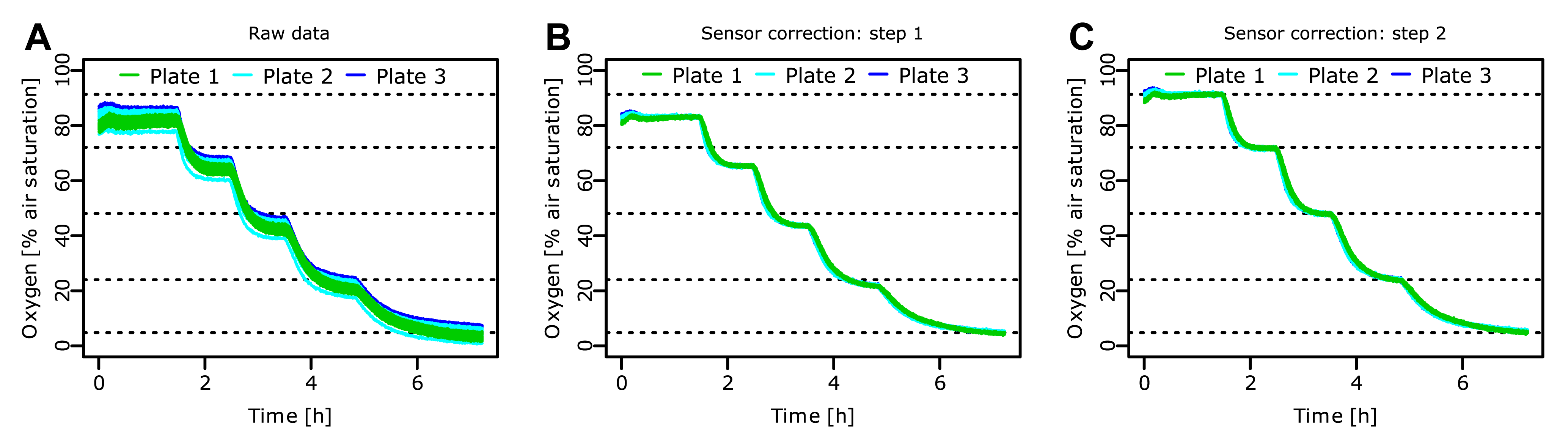

Supplement: S4 Fig — Read-out of 3 empty independent OxoDish, each containing 24 oxygen sensors, when placed in an oxygen-controlled incubator set consecutively at 91.3, 72.1, 48.1, 24.0 and 4.8% a.s. (dashed black horizontal lines). Using the TReCCA Analyser, the raw oxygen values given by all the sensors (Fig A) are made more homogeneous by step 1 of sensor correction: logarithmic recalibration (Fig B), and set to their target value by step 2 of sensor correction: linear normalisation (Fig C). (TIF) [file pone.0131233.s004.tif]

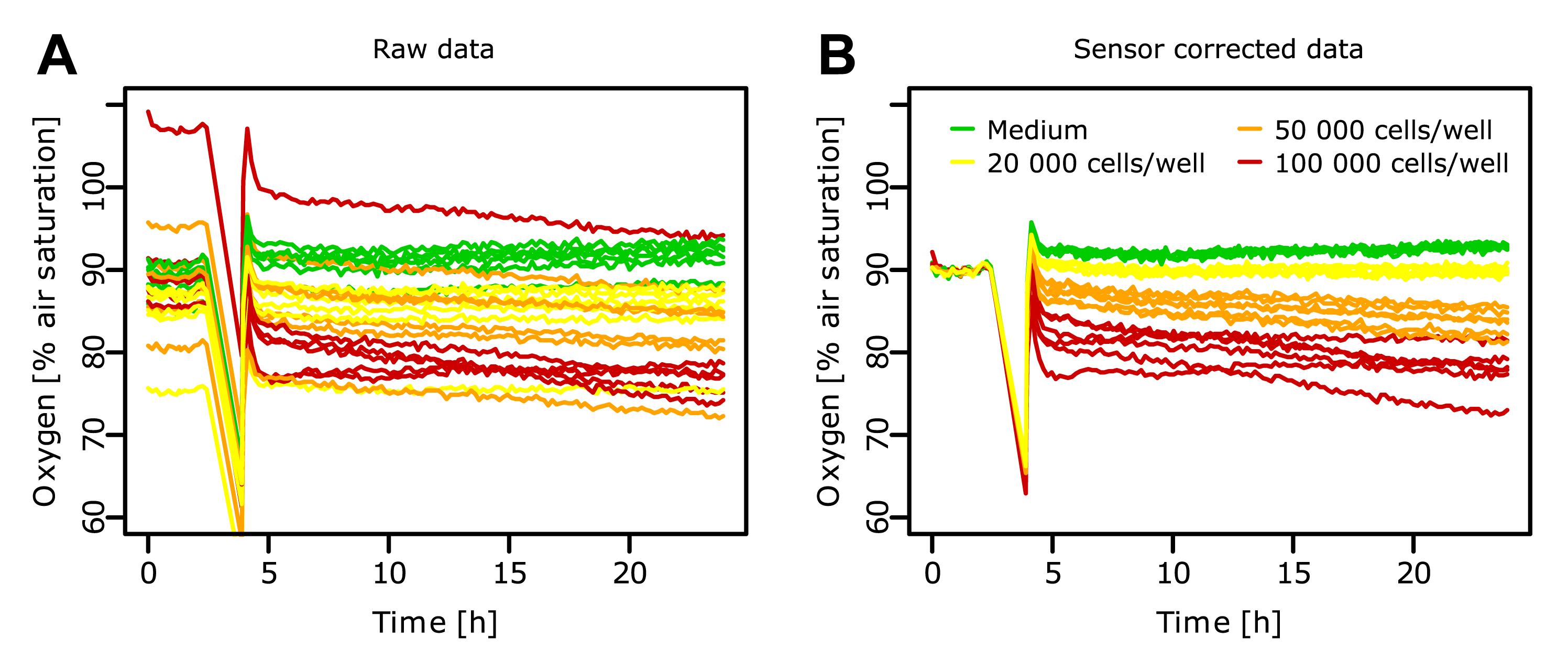

Supplement: S5 Fig — Oxygen level in the media of the Neuroblastoma cell line IMR5/75 at different seeding densities before (Fig A) and after sensor correction (Fig B). The legend in the Fig B in S5 Fig is valid for both subfigures. (TIF) [file pone.0131233.s005.tif]

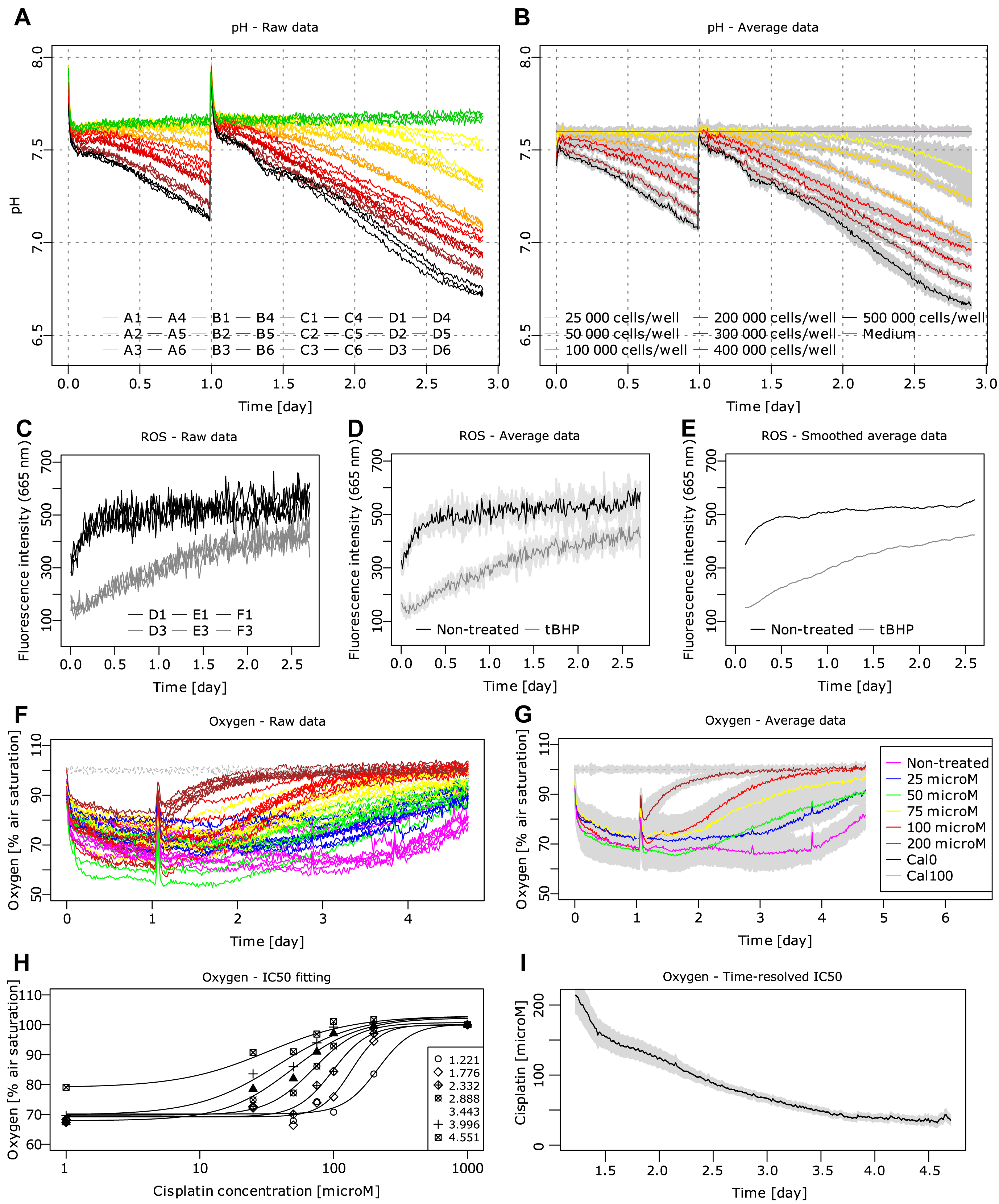

Supplement: S6 Fig — The pH of the medium of HT29 cells (ATCC HTB-38) cells seeded at varying seeding densities on day 0 was measured continuously using the SDR and HydroDish system (PreSens Precision Sensing GmbH, Germany) (Fig A). On day 1, the medium was changed in each condition setting the pH back to its original level. The data was normalised by setting the medium condition (containing no cells) to pH 7.6 and the technical triplicates were averaged (Fig B). The levels of ROS (Reactive Oxygen Species) were continuously measured in IMR5/75 cells when exposed to tBHP (tert-Butyl hydroperoxide). The cells were seeded in a 96-well plate at 20 000 cells/well and after 24 h they were treated and stained with 5 μM of CellROX Deep Red Reagent (Thermo Fischer Scientific Inc, United States). The fluorescence was continuously measured according to the recommendations of the dye provider using the Safire plate reader (Tecan Trading AG, Switzerland). The raw data (Fig C) was averaged (Fig D) and smoothed (Fig E) using the TReCCA Analyser. The level of dissolved oxygen in the medium of HT29 three-dimensional structures was continuously measured using a 96-well plate containing oxygen sensors in each well (OxoPlate, PreSens Precision Screening GmbH, Germany) and the Infinite 200 Pro plate reader (Tecan Trading AG, Switzerland). On day 1, half of the medium was exchanged and different concentrations of Cisplatin (Sigma-Aldrich LLC, United States) were added to each well in sextuplates. The raw data (Fig F) was normalised to the conditions with no cells and averaged (Fig G). The time-resolved IC50 was determined (Fig I) by continuously fitting the data using the TReCCA Analyser, as exemplified for seven time points (Fig H). (TIF) [file pone.0131233.s006.tif]
