# Supplementary material for: Time-Resolved Cell Culture Assay Analyser (TReCCA Analyser) for the Analysis of On-Line Data: Data Integration—Sensor Correction—Time-Resolved IC50 Determination
Source: PLoS One. 2015 Jun 25;10(6):e0131233. doi: 10.1371/journal.pone.0131233 (PMC4482264; doi:10.1371/journal.pone.0131233)
Supplement: S1 File — The supplementary equations are grouped in this file. (PDF) [file pone.0131233.s007.pdf]

Supplementary equations

A guide for the use of the program and more details about the formulas applied are available in the **TReCCA Analyser user manual**:
www.uni-heidelberg.de/fakultaeten/biowissenschaften/ipmb/biologie/woelfl/Research.html.
Two tutorials including the data used for this publication are also available at this address:

- **Tutorial 1** - Sensor correction, Normalisation, Averaging and IC50 determination
- **Tutorial 2** - Smoothing and slope calculation

Sensor correction: recalibration

Equation A: The mean of each sensor m_s is the average of the logarithmic data z of each sensor s for n calibration time points and a calibration time frame ranging from t_1 to t_n.

$$m_s = \frac{1}{n}\sum_{i=0}^{n-1} z_{t_{1+i},\ s}$$

Equation B: The mean of the whole plate m_p is the average of the mean of each sensor m_s for s number of sensors.

$$m_p = \frac{1}{s}\sum_{i=1}^{s} m_i$$

Sensor correction: normalisation

Equation C: The normalised data $y_{t,s}$ for each time point t and each sensor s is the data $x_{t,s}$ divided by the average of each calibrated sensor mc (Equation A in S1 File) and multiplied by the target value T.

$$y_{t,s} = \frac{x_{t,s} \cdot T}{mc_s}$$

Normalisation

Equation D: The normalised data y_t for each time point t is the data x_t divided by the average of the normalisation condition over time m_t and multiplied by the target value M.

$$y_t = \frac{x_t \cdot M}{m_t}$$

Average and standard deviation

Equation E: Standard deviation $sd(x)$, where $\bar{x}$ is the average and n the number of conditions averaged.

$$sd(x) = \sqrt{\frac{1}{n-1}\sum_{i=1}^{n}(x_i - \bar{x})^2}$$

Smoothing

Equation F: The smoothed data point y_t at the time point t is the average of the data point x_t and n neighbouring data points.

$$y_t = \frac{1}{n} \sum_{i=t-\frac{n-1}{2}}^{t+\frac{n-1}{2}} x_i$$

Slope calculation

Equation G: The slope m is determined by applying a linear fit function to each smoothed data point y_t and n smoothed data points on either side for their corresponding time points t.

$$(y_{x-n}, \cdots, y_{x+n}) = m \cdot (t_{x-n}, \cdots, t_{x+n}) + c$$

Time-resolved IC_{50}

Equations H, I and J: Two, three and five log-logistic model functions respectively. b is the slope of the hill, c and d are the upper and lower plateau respectively, e is the IC_{50} value and f differs from 1 if the function is asymmetric.

$$f(x,(b,e)) = \frac{1}{1 + e^{(b(\log(x) - \log(e)))}}$$

$$f(x,(b,d,e)) = \frac{d}{1 + e^{(b(\log(x) - \log(e)))}}$$

$$f(x,(b,c,d,e,f)) = \frac{d-c}{\left(1 + e^{(b(\log(x) - \log(e)))}\right)^f} + c$$
