# Supplementary material for: Time-Resolved Cell Culture Assay Analyser (TReCCA Analyser) for the Analysis of On-Line Data: Data Integration—Sensor Correction—Time-Resolved IC50 Determination
Source: PLoS One. 2015 Jun 25;10(6):e0131233. doi: 10.1371/journal.pone.0131233 (PMC4482264; doi:10.1371/journal.pone.0131233)
Supplement: S2 File — Details about the sensor correction method in the particular example of OxoDish use are presented in this file. (PDF) [file pone.0131233.s008.pdf]

Sensor correction for OxoDish

The development and justification of a sensor correction method to homogenise the initial output of the OxoDish sensors is presented here. First the experimental design is detailed, followed by the two steps of sensor correction: sensor recalibration and normalisation.

Experimental design for the OxoDish sensor correction method

The oxygen was measured in percentage of air saturation (a.s.) and the lot number of the OxoDish OD-1133-01 was entered into the SDR's software for the general calibration of the plates. The measuring interval was here set to 15 s. All the 24 sensors of 3 independent OxoDish were measured without any content in each well at varying oxygen saturation levels: 19, 15, 10, 5 and 1 % oxygen saturation which converts to 91.3, 72.1, 48.1, 24.0 and 4.8 % a.s. by taking into account the average oxygen saturation of the laboratory air of 20.8 %. This was performed in an oxygen and humidity controlled incubator with 5 % CO_2 and at 37 °C (Heracell™ 240i, Thermo Fisher Scientific Inc., United States). The oxygen values given by the incubator were controlled at the end of the experiment by using oxygen Sensor Spots read-out by a Fibox 3 (PreSens Precision Sensing GmbH, Germany).

Sensor recalibration

For each OxoDish, the standard deviation of the read-out of the 24 sensors was determined for each time point and was used to evaluate the homogeneity of the plate output. The average standard deviation of the last 10 time points of each oxygen condition (at 1.5, 2.5, 3.5, 4.8 and 7.2 hours) was then calculated to represent the homogeneity depending on the oxygen saturation.

The read-out at the initial 91.3 % a.s. was averaged for each sensor over the 10 last time points, thus giving a representative start value for each sensor. Then, these initial average values were averaged themselves to determine the altogether read-out of the OxoDish. The initial average of each individual sensor was then set to the average of the sensor dish, using linear correction with or without logarithmic conversion (Figure A in S3 Fig.). Linear correction (dividing the data of each sensor by the sensor average and then multiplying it by the sensor plate average) homogenises the data for high oxygen levels but not for low oxygen levels. The homogenisation of the sensor data on all the oxygen levels is obtained by going through a logarithmic conversion, then correcting the data linearly using the also logarithmically converted averages, and finally exponentially converting the data back to their corrected non logarithmic form. The standard deviation between the sensors was thereby lowered from 0.96 to 0.23 % a.s. at 91.3 % a.s. and from 0.78 to 0.17 % a.s. at 4.8 % a.s. compared to the original data.

This result implies an exponential relationship between the phase angle values and the oxygen concentration, which is surprising as they should be linked through a Stern-Volmer relationship [14]. The exact calibration formula being the property of PreSens Precision Sensing GmbH, we fitted 60 calibration points provided by the company using Prism (GraphPad Software, Inc, United States) with the model $P + Se^{\alpha y}$, where P is the plateau, S the span and α an unknown factor. Between 5 and 100 % a.s., the average percent error between the actual data points and the exponential model equation was found to be 0.25 % for three independent lots and curve fittings, confirming the use of this model. The span and

plateau values for the different OxoDish lots can be found in the TReCCA Analyser user manual.

As a control, this function was applied to the data (Figure A and B in S4 Fig.) where the reduction of the standard deviation of the read-out of the 24 sensors is clearly visible, without the average level being modified.

Sensor normalisation

The read-outs of the 72 sensors were compared to each other and to the incubator oxygen value after they had been recalibrated.

The off-sets of the three independent OxoDish after sensor recalibration was found to be linearly proportional to the oxygen saturation (Figure B in S3 Fig.) so the data was corrected further by multiplication. First, the average of each calibrated sensor *mc* is calculated over *n* calibration time points (Equation A in S1 File) and then all the data is divided by *mc* and multiplied by a target value *T* (Equation C in S1 File). When applied to the already recalibrated data (Figure B and C in S4 Fig.), the sensor normalisation sets the measured values to the actual values.
